# Effect of Endogenous Arginine-Vasopressin Arising from the Paraventricular Nucleus on Learning and Memory Functions in Vascular Dementia Model Rats

**DOI:** 10.1155/2017/3214918

**Published:** 2017-11-28

**Authors:** Chun-Ying Li, Lei Zhang, Jing Li, Chun-Li Qi, Dong-Ying Li, Xu Liu, Xian Qu

**Affiliations:** ^1^Department of Physiology, Basic Medical College of Beihua University, Jilin 132013, China; ^2^Nursing College of Beihua University, Jilin 132013, China; ^3^Institute of Laboratory Animal Science, Jinan University, Guangzhou, Guangdong 510632, China; ^4^Sixth People's Hospital of Jilin City, Jilin 132000, China

## Abstract

The hippocampus is a key structure for encoding and processing memory and for spatial orientation, which are among the cognitive functions most sensitive to cerebral ischemia, hypoxia, and vascular dementia (VD). Since hippocampal formation is one of the principle forebrain targets for arginine-vasopressin (AVP) innervations arising in the hypothalamic paraventricular nucleus (PVN), we explored the contributions of AVP to VD pathogenesis. To this end, we randomly assigned pathogen-free, male Wistar rats to one of seven groups in a VD model and tested AVP treatment effects on spatial learning and memory using the Morris water maze. We also measured the superoxide dismutase (SOD) activity and malondialdehyde (MDA) concentration in brain samples and monitored the expression of AVP-positive neurons in the hippocampus by immunohistochemistry. The VD model with repeated cerebral ischemia-reperfusion injury evoked impairment of cognitive function and reduced cerebral concentrations of the antioxidation markers. Lesioning the rat PVN showed a similar effect on learning and memory and reduced antioxidation markers in the brain tissue. However, AVP injection into the PVN improved cognitive performance in VD rats, while enhancing/rectifying the changes in antioxidation markers. We conclude that our VD model may decrease AVP secretion in the PVN and subsequently reduce antioxidant capacity in the hippocampus, leading to impaired cognitive function.

## 1. Introduction

The pathogenesis of cerebrovascular diseases had been linked to diverse factors, including central cholinergic system dysfunction, increased generation of oxygen free radicals, neuroinflammation, amyloid-*β* deposition, and apoptosis [1–3]. Hippocampal formation, which plays a key role in the consolidation and retrieval of episodic and spatial memory, is among the brain structures most vulnerable to cerebral ischemia [4], and impaired hippocampal function is an important factor in the cognitive dysfunction of Alzheimer's disease (AD). Vascular dementia (VD) is (after AD) the next most common cause of cognitive dysfunction and is associated with hippocampal damage [5–12].

Tract-tracing experiments have revealed direct fiber connections from magnocellular neurons of the hypothalamic paraventricular nucleus (PVN) to the hippocampus [13], with reciprocal projections from the hippocampus back to the PVN [14]. The magnocellular neurons of the PVN and nearby suprachiasmatic nucleus express high levels of the neuromodulatory peptide arginine-vasopressin (AVP) and give rise to a hippocampal AVP innervation ascending via the fornix and (to the ventral hippocampus) via the corpus fimbriatum. Indeed, hippocampal formation is one of the principle forebrain targets for AVP innervations, and it exerts a descending control of AVP secretion.

Within the hippocampus, AVP can act on the dentate gyrus, as shown by lesion and microinjection studies, and hippocampal stimulation alters AVP mRNA expression in PVN neurons, supporting a reciprocal relationship between hippocampal function and the AVP-positive hypothalamic neurons. An extensive body of evidence shows that AVP can strengthen learning and memory functions and facilitate recall [15–20]. Given this association, we wished to determine whether endogenous AVP arising from the PVN is a factor in the pathophysiology of VD. Therefore, we studied the effect of PVN lesions and AVP microinjections on intact PVN in learning and memory functions in a rat VD model.

## 2. Materials and Methods

### 2.1. Animals

Specific pathogen-free (SPF) male Wistar rats (*N* = 75) aged 10–12 weeks were obtained from the Laboratory Animal Center of Jilin University, China (certification number SCXK (Ji) 2007-0003). Animals were housed at standard temperature (22 ± 1°C), humidity (40–50%), and light-controlled conditions (12 h light/dark cycle). All animals acclimatized in the facility under these conditions for one week prior to the experiments. This experiment was approved by the Animal Ethics Committee of Jilin University and performed in accordance with Decree number 2 of the State and Technology Commission of China (approved by the State Council on October 31, 1988, and promulgated by the State Science and Technology Commission on November 14, 1988).

Groups of 10 rats were randomly assigned to one of the following seven groups: (1) normal controls, without any particular treatment; (2) sham-operation controls, entailing surgical isolation of the common carotid artery, with insertion of silk, but no obstruction of blood flow; (3) VD model; (4) daily artificial cerebrospinal fluid (ACSF 0.2 *μ*L) infusion beginning 2 weeks after VD and continuing for 1 week; (5) AVP infusion (600 ng in 0.2 *μ*L ACSF) beginning 2 weeks after VD and continuing for 1 week; (6) sham-lesioned group, with placement of an inactive electrode in the PVN; and (7) PVN-lesioned group, with electrical lesion of the PVN in otherwise normal rats. We had an ACSF (vehicle) experimental group serving as a control group for the AVP group in order to accommodate possible effects of the vehicle. Due to loss of five animals at the time of surgery, we required 75 animals to obtain final group sizes of 10 animals.

### 2.2. Surgery in the Vascular Dementia Model with Cannula Insertion

Animals were anesthetized by intraperitoneal injection of 10% chloral hydrate (3.5 mL/kg body weight), were placed in a prone position, and received a neck incision under sterile conditions. VD model rats received an injection of sodium nitrate solution (2.5 mg/kg, i.p.) to reduce blood pressure.

After surgical isolation of the bilateral carotid artery by blunt dissection, sutures were placed about the arteries for three successive cycles of bilateral carotid artery blockade (each lasting 10 min), followed by reperfusion for 10 min. For stereotaxic lesions, anesthetized rats were placed in the stereotaxic instrument (Narishige SR-9, Tokyo, Japan), and the skull was exposed under sterile conditions.

Following the coordinates of the Paxinos and Watson Stereotaxic Atlas [21], a stainless steel guide cannula was placed in the bilateral PVN (anterior-posterior −1.08 mm, lateral 0 mm, ventral 7.0 mm). After placement, the cannula was fixed to the skull with dental cement, and rats received a prophylactic intraperitoneal antibiotic treatment after scalp suturing.

### 2.3. Microinjection

AVP infusions were made to the midline VPN through the guide cannula using an injection needle (RWD Life Science Co., Ltd., Shenzhen, China) connected by polyethylene tubing to a microsyringe (Baoding LanGeHeng Co., Ltd., Baoding City, China). The injection needle replaced the stylet, with the tip extending 0.2 mm beyond the end of the guide cannula. After 0.2 *μ*L intracerebral infusions, the needle remained in place for 1 minute and was then replaced by the stylet to block the guide cannula.

### 2.4. Paraventricular Nucleus Lesions

Animals were anesthetized and placed in the stereotaxic frame as described above. A chronic indwelling (in-house manufactured) electrode was placed in the PVN at the coordinates described above, and a destructive lesion was obtained by application of direct current (1 mA, 1 min; Lesion Making Device #53500, UGO Basile, Varese, Italy).

### 2.5. Morris Water Maze

The Morris water maze consisted of a circular water tank of 120 cm diameter and 50 cm depth divided into four equal quadrants designated A, B, C, and D. A platform of 10 cm diameter was fixed in one quadrant, lying 1.5 cm below the water surface. Over a period of five days, rats were trained to find the platform, and their latencies (time required to reach the platform) were recorded in each trial; rats were rescued if they failed to find the platform within 120 sec. The platform was removed after the five-day trial, and the time and number of crossings to the platform position were recorded on the sixth day.

### 2.6. Measurement of Superoxide Dismutase Activity and Malondialdehyde Concentration

Animals were killed after being anesthetized as above, and their brains were rapidly removed for dissection of the bilateral hippocampi. Tissue was homogenized in 10 volumes of ice-cold PBS buffer using a glass-Teflon homogenizer. The homogenate was centrifuged for 10 min at 3000 rpm, and the supernatant was stored at −20°C. The superoxide dismutase (SOD) activity and the malondialdehyde (MDA) concentration were measured using a commercial kit (Nanjing Jiancheng Bioengineering Institute, Nanjing, China).

### 2.7. Immunohistochemical Analysis

Animals were anesthetized as above and perfused with normal saline followed by a solution of 4% paraformaldehyde (PFA) in 0.1 M PBS (pH 7.2–7.4). After perfusion, coronal plane blocks containing the hippocampus were postfixed in 4% PFA for 24 h, dehydrated in a series of ethanol solutions of increasing concentration, and embedded in paraffin. The paraffin-embedded blocks were then cut into 2 *μ*m thick sections and processed for AVP immunohistochemistry by incubation in rabbit anti-rat AVP primary antibody (1 : 500 dilution in PBS; Abnova PAB7839; Abnova, Taipei City, Taiwan) for 24 h at room temperature.

Immunostaining was detected using an UltraSensitive™ SP kit according to the manufacturer's instructions (KIT-9710, Fuzhou Maxim Inc., Fujian, China). In brief, sections were washed with PBS and labeled with a biotin-labeled secondary anti-rabbit antibody (Solution C, 50 *μ*L) and streptavidin-anti-biotin-peroxidase (Solution D, 50 *μ*L). Immunostaining was visualized using a 3,3′-diaminobenzidine tetrahydrochloride (DAB) kit (DAB-0031; Fuzhou Maxim Inc., Fujian, China). Sections were then counterstained with hematoxylin and eosin, washed with running tap water, dehydrated in an ethanol series, cleared in xylene, and mounted on glass slides with neutral balsam. Based on their laminar distribution and the known cytoarchitecture of the hippocampus, we are certain that these are pyramidal neurons. The mean intensity of AVP staining in sections was determined by analysis of randomly selected fields and quantification with IPP v6.0 software (Media Cybernetics, Silver Spring, MD, USA).

### 2.8. Statistical Analysis

Statistical analysis was performed with the SPSS v16.0 software (SPSS, Chicago, IL, USA) and presented as mean ± standard deviation (SD). Statistical significance was determined using one-way analysis of variance (LSD ANOVA posttest). *P* < 0.05 was considered statistically significant.

## 3. Results

### 3.1. Results of Morris Water Maze Test

#### 3.1.1. Searching Platform Testing

**Figure 1:**
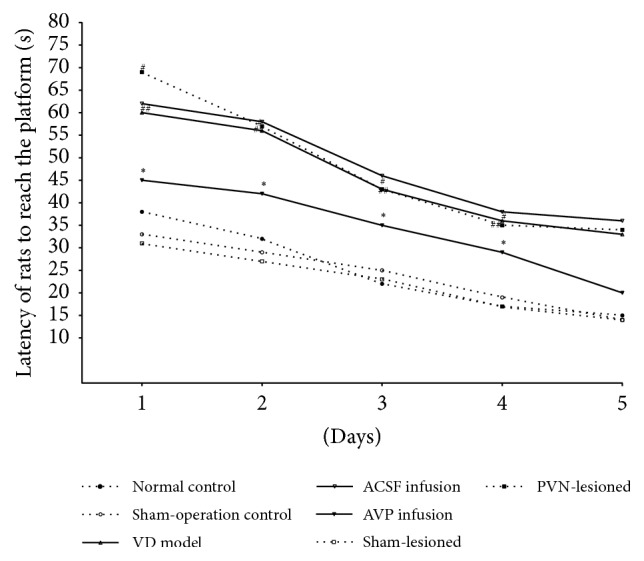
Latency of rats to reach the platform of the Morris water maze. *n* = 10 animals in each group. ^##^*P* < 0.05 versus normal control group; ^*∗*^*P* < 0.05 versus ACSF infusion group; ^#^*P* < 0.05 versus sham-lesioned group.

With increasing training time, the latency to reach the platform was significantly reduced in all groups. Compared with the normal control group, the latencies did not differ in the sham-operation control group and sham-lesioned group (*P* > 0.05) but were significantly longer in the VD model group (*P* < 0.05). There was no difference in latency between the VD model and ACSF infusion groups, but latency was significantly reduced in the AVP infusion group (*P* < 0.05). Latency was significantly increased (i.e., more time taken to perform the task) in the PVN-lesioned group relative to the sham-lesioned group (*P* < 0.05) (Figure 1).

#### 3.1.2. Spatial Exploration Testing

**Figure 2:**
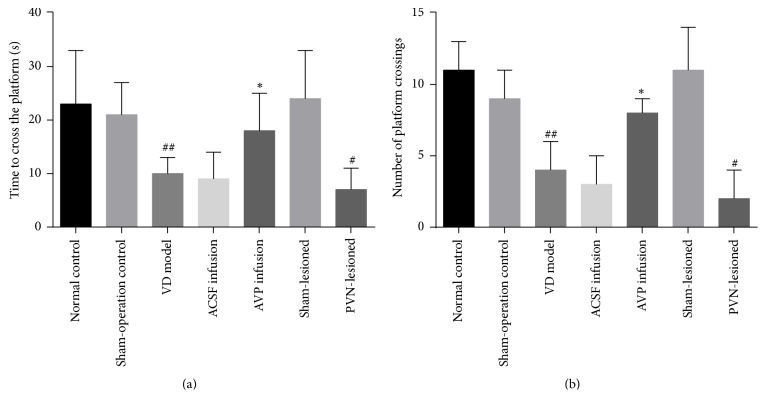
Spatial exploration testing in rats in the VD model. (a) *n* = 10 animals in each group. ^##^*P* < 0.05 versus normal control group; ^*∗*^*P* < 0.05 versus ACSF infusion group; ^#^*P* < 0.05 versus sham-lesioned group. (b) *n* = 10 animals in each group. ^##^*P* < 0.05 versus normal control group; ^*∗*^*P* < 0.05 versus ACSF infusion group; ^#^*P* < 0.05 versus sham-lesioned group.

Compared with the normal control group, time to cross the platform and number of platform crossings did not differ in the sham-operation control and sham-lesioned groups (*P* > 0.05) but were significantly decreased in the VD model group (*P* < 0.05). Scores did not differ between the VD model and ACSF infusion groups but were significantly higher in the AVP infusion group relative to the ACSF infusion group (*P* < 0.05). Relative to the sham-lesioned group, times were significantly higher in the PVN-lesioned group (*P* < 0.05) (Figures 2(a) and 2(b)).

### 3.2. Superoxide Dismutase Activity and Malondialdehyde Concentration in the Hippocampus

**Figure 3:**
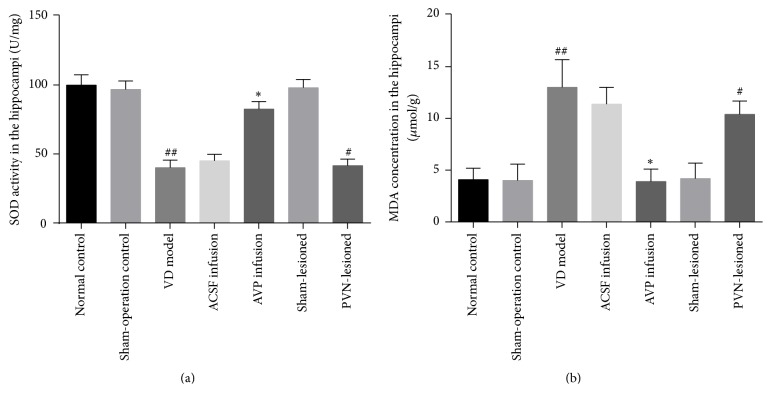
SOD activity and MDA concentration in the hippocampi of rats in the following groups. (a) *n* = 10 animals in each group. ^##^*P* < 0.05 versus normal control group; ^*∗*^*P* < 0.05 versus ACSF infusion group; ^#^*P* < 0.05 versus sham-lesioned group. (b) *n* = 10 animals in each group. ^##^*P* < 0.05 versus normal control group; ^*∗*^*P* < 0.05 versus ACSF infusion group; ^#^*P* < 0.05 versus sham-lesioned group.

Compared with the normal control group, the SOD activity and MDA concentration in the hippocampus did not differ in the sham-operation control and sham-lesioned groups (*P* > 0.05), but the SOD activity was significantly lower in the VD model group, whereas the MDA concentration was significantly higher (*P* < 0.05). There were no such differences between the VD model and ACSF infusion groups, but compared with the ACSF infusion group, the SOD activity was higher and MDA concentration was lower in the AVP infusion group (*P* < 0.05). Compared with the sham-lesioned group, the SOD activity was higher and the MDA concentration was lower in the PVN-lesioned group (*P* < 0.05) (Figures 3(a) and 3(b)).

### 3.3. Arginine-Vasopressin-Positive Neurons in the Hippocampal Region

Since the CA1 is well known as the hippocampal region most sensitive to cerebral ischemia, we chose to analyze this region. The expression of AVP-positive neurons in the hippocampal region was elevated, with deeper immunostaining in the normal control, sham-operation control, and sham-lesioned groups than in the VD groups. The AVP-positive neurons were hypochromatic, with narrow soma and with sparser distribution in PVN-lesioned animals.

**Figure 4:**
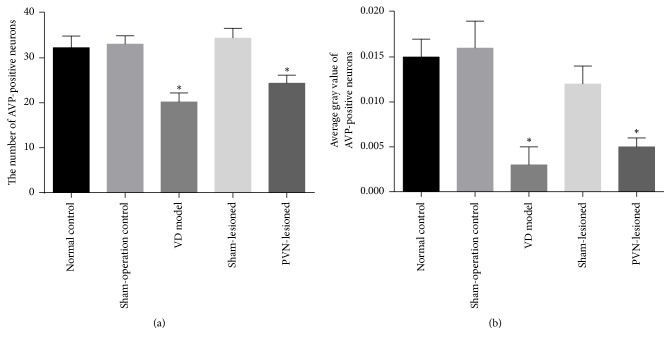
The number and average gray value of AVP-positive neurons in the hippocampal region. (a) *n* = 10 animals in each group. (b) *n* = 10 animals in each group. ^*∗*^*P* < 0.05 versus normal control group.

**Figure 5:**
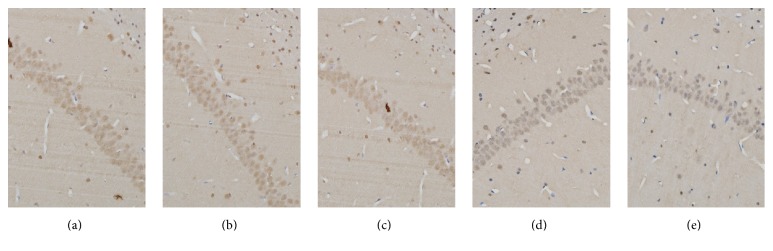
The expression of AVP-positive neurons in the hippocampal region (immunohistochemistry, 200x): (a) normal control group, (b) sham-operation group, (c) sham-lesioned group, (d) PVN-lesioned group, and (e) VD model group.

Compared with the normal control group, the number and the average gray scale intensity value of AVP-positive neurons in the hippocampal region did not significantly differ in the sham-operation control and sham-lesioned groups. However, the number and the average gray value of AVP-positive neurons were distinctly decreased in the PVN-lesioned group (*P* < 0.05) (Figures 4(a), 4(b), 5(a), 5(b), 5(c), 5(d), and 5(e)).

## 4. Discussion

The capacity for learning and memory is a defining characteristic of organisms with a highly developed nervous system, and this capacity is indispensable for human survival. As in other forms of dementia, a main clinical manifestation among patients is a significant decline in cognitive function, including deficits in spatial learning and memory. The Morris water maze is an important research tool for assessing subtle deficits in spatial learning and memory in rodent models of VD and other models of cognitive impairment [22]. An advantage of the Morris water maze lies in its minimization of olfactory confounds arising from stool and hormone deposition in ordinary mazes [23]. Our results showed significant impairment of learning and memory functions in VD rats, which is consistent with reports in the literature [24].

AVP derived from magnocellular neurons of the hypothalamus has neurotransmitter or neuromodulator functions in addition to its classical neuroendocrine role in regulation of water balance via release from the hypophysis. These additional functions of AVP relate to diverse behaviors and homeostatic mechanisms, such as learning and memory, social behavior, circadian rhythm, thermoregulation, and autonomic function. In keeping with these actions, receptors for AVP are abundant in various brain regions, such as the hippocampus, amygdala, and lateral hypothalamus [25–27].

Research has confirmed that, after ablation of AVP neurons of the posterior pituitary, learning is impaired in rats, whereas treating such rats with AVP can restore memory function via a central action [28]. Hippocampal formation serves important functions in learning and memory performance [29, 30], which are highly sensitive to ischemia and hypoxia [4]. AVP transmission in the ventral hippocampus may be involved in information processing, storage, and retrieval, especially for memory consolidation [31]. Given that the functional state and the number of hippocampal neurons are important factors in synaptic plasticity relevant to learning, AVP emerges as a potential mediator of cognitive and neurochemical effects seen in the present VD model of memory dysfunction.

Increased generation of free radicals occurs in several acute or chronic injuries of the brain, including ischemic brain injury. Free radicals produce oxidative damage, which can lead to neuronal loss in vulnerable brain regions such as the hippocampus [32]. The SOD enzyme plays an important role in the regulation of the oxidative and antioxidative balance in the brain tissue, thus providing an indicator of the tissue's ability to remove free radicals.

As the concentration of MDA is an indicator of oxidative stress, SOD enzyme serves as an additional biomarker in the pathology of neurodegenerative diseases [33]. We find that SOD activity decreased while MDA concentration increased in the hippocampal formation of our VD model rats and that these changes were unaffected by ACSF infusion into the hypothalamus. We propose that abnormal free-radical metabolism in the hippocampus of VD rats is plausibly linked to their impaired spatial memory performance.

The AVP expression in the hippocampi of our VD rats was conspicuously lower in immunohistochemistry. Furthermore, the reduced hippocampal SOD activity and increased MDA concentration in VD rats normalized with the AVP infusion, thus supporting a link between oxidative status, AVP transmission, and spatial memory. We conclude that hippocampal AVP innervations arising in the hypothalamus have an action leading to improved free-radical metabolism in the stressed brain, which may mediate rescue from cognitive effects arising from the VD model. Of course, the hypothalamic-hippocampal pathway need not be the only relevant factor in mediating cognitive deficits in the present VD model.

Our present results show that decreased secretion of AVP from PVN neurons may be a factor in the pathophysiology of VD, propagating to reduced AVP content and antioxidant capacity in the hippocampus. In addition, our results imply that treatment with AVP peptides or small-molecule agonists may present a new therapeutic avenue in the treatment of VD.
